# Spontaneous Chylothorax following Septic Pulmonary Embolization

**DOI:** 10.1155/2020/3979507

**Published:** 2020-02-21

**Authors:** Michael Agustin, Michele Yamamoto, Chawat Tongma, Leslie Anne Chua, Michael Torres, Scott Shay

**Affiliations:** Guam Regional Medical City, 133 Route 3, Dededo Guam, USA 96929

## Abstract

Chylothorax is the occurrence of chyle (lymph) in the pleural cavity secondary to damage of the thoracic duct. It is a rare form of pleural effusion which appears as a milky white turbid fluid. Malignancy is the leading cause of nontraumatic chylothorax while inadvertent surgical injury to the thoracic duct is the major cause of traumatic chylothorax. We report a case of spontaneous left-side chylothorax following septic pulmonary embolization (SPE) with Methicillin-Resistant Staphylococcus aureus (MRSA). This is a rare case of a nonmalignant, nontraumatic, and nontuberculous spontaneous chylothorax which was conservatively treated with fibrinolysis and diet modification.

## 1. Introduction

The accumulation of lymph in the pleural space due to damage or obstruction of the thoracic duct results in chylothorax. It is an uncommon cause of pleural effusion with high content of triglycerides. The presence of chylomicrons in the pleural fluid is the definitive diagnostic criterion of chylothorax [1]. Although nontraumatic etiology of chylothorax is mostly related to malignancy, the role of infection leading to thoracic duct damage has not been reported in the literature. Not only does septic pulmonary embolism present with pulmonary cavitation but also a third of patients affected may present with pleural effusion on chest imaging [2]. Empyematous chylothorax is a rare phenomenon that is linked to tuberculosis in a case report [3]. We present a rare sequela of severe septic pulmonary embolization causing chylothorax with possible coexisting empyema.

## 2. Case Presentation

**Figure 1:**
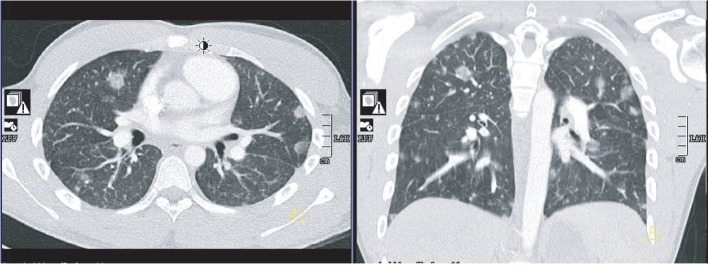
Chest computed tomography (CT) on admission showing bilateral pulmonary nodules consistent with septic pulmonary embolization (SPE) in the setting of Methicillin-Resistant Staphylococcus aureus (MRSA) bacteremia.

**Figure 2:**
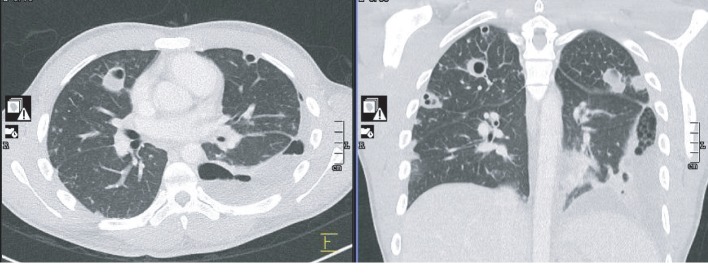
Repeat chest computed tomography (CT) on hospital day twelve (12) showing more pronounced cavitary lesions and moderate loculated left lower lobe pleural effusion.

**Figure 3:**
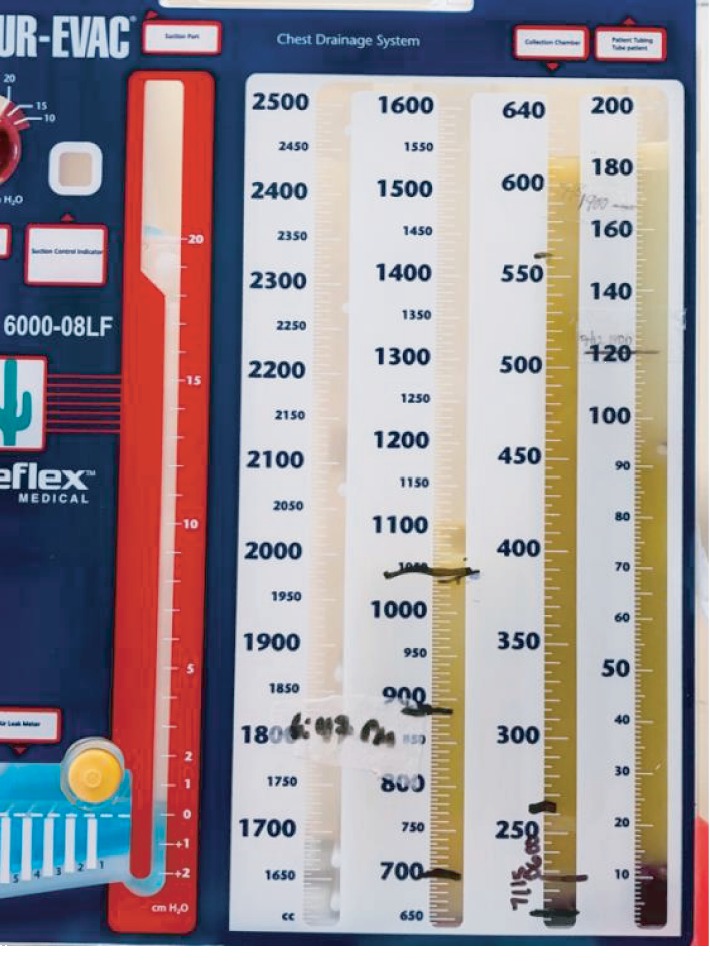
Pleural fluid from left chest drainage with milky appearance consistent with chylothorax.

**Figure 4:**
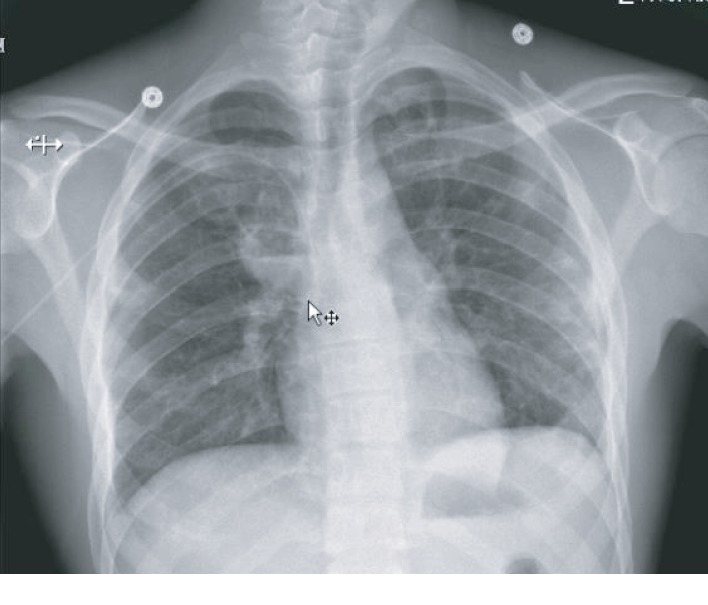
Chest X-ray showing interval improvement of left chylothorax 10 days after intrapleural fibrinolytics and diet modification.

An eighteen-year-old previous healthy male was admitted for worsening chest pain, cough, and dyspnea. Chest computed tomography (CT) (Figure 1) showed bilateral cavitary lesions with no mediastinal hilar adenopathy and no pleural effusion. There was no pulmonary artery filling defect or cardiac filling defect. We did not see any symptoms suggestive of jugular or subclavian vein thrombosis to warrant dedicated imaging. The patient was found to have MRSA bacteremia which likely originated from a draining pilonidal cyst. The patient was ruled out of bacterial endocarditis, and Mycobacterium tuberculosis studies were all negative. There is no other pertinent embolic or mass-like lesion in the abdomen and brain. Twelve days through the course of antibiotics, repeat chest imaging showed more pronounced diffuse cavitary lesions now accompanied by left-sided loculated pleural effusion (Figure 2). The patient also presented with worsening peripheral eosinophilia. Milky pleural fluid of about 300 mL was obtained on initial pleural drainage (Figure 3) which was mixed with some blood. Pleural fluid analysis showed RBC of 81111 cells/*μ*L, WBC of 444 cells/*μ*L with 65% segmenters, pleural fluid pH of 8, LDH of 659 IU/L, pleural fluid protein of 6 g/dL, and glucose of 28 mg/dL. The pleural fluid/serum LDH ratio is 4.2 and the fluid/serum protein ratio is 1, which is thus reflective of exudative effusion. Chylothorax was confirmed with pleural fluid triglyceride of 319 mg/dL and cholesterol of 84 mg/dL. There was no microbiologic growth on the pleural fluid. The possibility of an infected chylothorax was considered given the bacteremia and exudative nature of pleural fluid. The team decided for fibrinolysis with two (2) doses of 4 mg Alteplase instilled intrapleurally due to the loculated nature of the effusion. In addition to fibrinolytic treatment, the patient was placed on low-fat with medium-chain triglyceride and high-protein diet. Fluid cytology was negative for malignancy, and all serologies to rule out connective tissue disease were negative. Strongyloides IgG was negative. Over time, pleural drainage decreased and the chest tube was removed after 5 days with no pleurodesis required. There was interval improvement of chest imaging ten (10) days postfibrinolytics (Figure 4).

## 3. Discussion

The accumulation of lymph in the pleural space due to damage or obstruction of the thoracic duct results in chylothorax. It is an uncommon cause of pleural effusion with high content of triglycerides, and the presence of chylomicrons is the definitive diagnostic criterion of chylothorax [1]. In a large single-center case series, about 50% of the causes of chylothorax are surgery or trauma-related. The medical and idiopathic causes of chylous pleural leakage are about 44% and 6%, respectively [4]. Among medical conditions, lymphoma, lymphatic disorders, and chylous ascites were the most common causes [4].

Chylothorax resulting from septic pulmonary embolism has not been reported in the literature. In our case, MRSA bacteremia led to septic pulmonary embolism. Pulmonary effusion and pulmonary abscess may be seen in cases of septic pulmonary embolization (SPE). In a systematic review of patients with SPE, pleural effusion is seen on 32% by X-ray and 29% by chest computed tomography (CT) imaging [2]. In another case series of SPE requiring critical care, the presence of pleural effusion is seen in 65% of cases while pulmonary abscess was noted in 30% [5]. We hypothesized that the extensive and diffuse cavitary lesions may have damaged the pulmonary lymphatic duct which led to leakage of chyle into the pleural space. We ruled out any other mediastinal obstruction that may have led to chyle leakage. As mentioned, about 6% of causes of chylothorax may be idiopathic [4]. In one case report, a temporal relationship between coughing and appearance of lymphatic effusion has been described with neck hyperextension as the likely etiology [6]. In a systematic review, cough may happen to 41% patients with spontaneous pulmonary embolization [2].

Chylous pleural effusions are typically described exudative lymphocytic pleural effusions with milky appearance. In a retrospective study, chylothoraces may present with variable pleural fluid appearance and biochemical characteristics [7]. The chylous pleural fluid appeared milky in only 44% and predominantly exudative (86%) [7]. The pleural fluid in our case was an exudative milky-appearing fluid.

Empyema was considered a differential diagnosis in our case given the loculated appearance of fluid on chest imaging. The pleural fluid's milky appearance, elevated fluid triglyceride, unremarkable pleural fluid pH, and WBC made us consider chylothorax the predominant pathology. The rare coexistence of empyema and chylothorax, known as empyematous chylothorax, is possible in this case given the overlapping biochemistry of the pleural fluid. In small case series, pleural fluid triglycerides in the chyliform range in the setting of acute bacterial parapneumonic effusion and empyema may indicate severity of the disease [8].

The treatment approach to chylothorax varies in that some clinicians adopt early surgical intervention while others adopt a conservative approach. Though the conservative approach may have a role to play in small chylothoraces, therapeutic thoracentesis or chest tube drainage is the initial step in large symptomatic chylothoraces. The initial output for this case was about 300 mL only; thus, our team has elected to drain the tube and modify the diet. Conservative treatment includes the use of a low-fat diet supplemented with medium-chain triglycerides (MCT) and/or total parenteral nutrition (TPN) [1].

Since our repeat CT of the chest showed evidence of loculated pleural fluid, two (2) doses of Alteplase were instilled intrapleurally. The role of fibrinolytics is not established but has been proposed for patients with idiopathic chylothorax who failed conservative therapy but refused surgery in one case report [9]. There were very limited case reports on the successful use of fibrinolytics for idiopathic chylothorax and empyematous multiloculated chylothorax [3, 9]. In conclusion, we report a rare case of nontraumatic, nonmalignant, and nontuberculous chylothorax which was considered a complication of severe pulmonary septic embolization that likely damaged the pulmonary lymphatic drainage. The possibility of an empyematous chylothorax in this case cannot be entirely ruled out. Conservative treatment with diet modification and intrapleural fibrinolytics resulted in clinical and radiographic improvement of this case.
